# Phytohormone and Light Regulation of Chlorophyll Degradation

**DOI:** 10.3389/fpls.2017.01911

**Published:** 2017-11-06

**Authors:** Xiaoyu Zhu, Junyi Chen, Kai Qiu, Benke Kuai

**Affiliations:** ^1^State Key Laboratory of Genetic Engineering and Fudan Center for Genetic Diversity and Designing Agriculture, School of Life Sciences, Fudan University, Shanghai, China; ^2^Ministry of Education, Key Laboratory for Biodiversity Science and Ecological Engineering, Institute of Biodiversity Science, Fudan University, Shanghai, China

**Keywords:** chlorophyll degradation, phytohormone, ethylene, abscisic acid, jasmonic acid, light

## Abstract

Degreening, due to the net loss of chlorophyll (Chl), is the most prominent symptom during the processes of leaf senescence, fruit ripening, and seed maturation. Over the last decade or so, extensive identifications of *Chl catabolic genes* (*CCGs*) have led to the revelation of the biochemical pathway of Chl degradation. As such, exploration of the regulatory mechanism of the degreening process is greatly facilitated. During the past few years, substantial progress has been made in elucidating the regulation of Chl degradation, particularly via the mediation of major phytohormones' signaling. Intriguingly, ethylene and abscisic acid's signaling have been demonstrated to interweave with light signaling in mediating the regulation of Chl degradation. In this review, we briefly summarize this progress, with an effort on providing a framework for further investigation of multifaceted and hierarchical regulations of Chl degradation.

## Introduction

**Figure 1:**
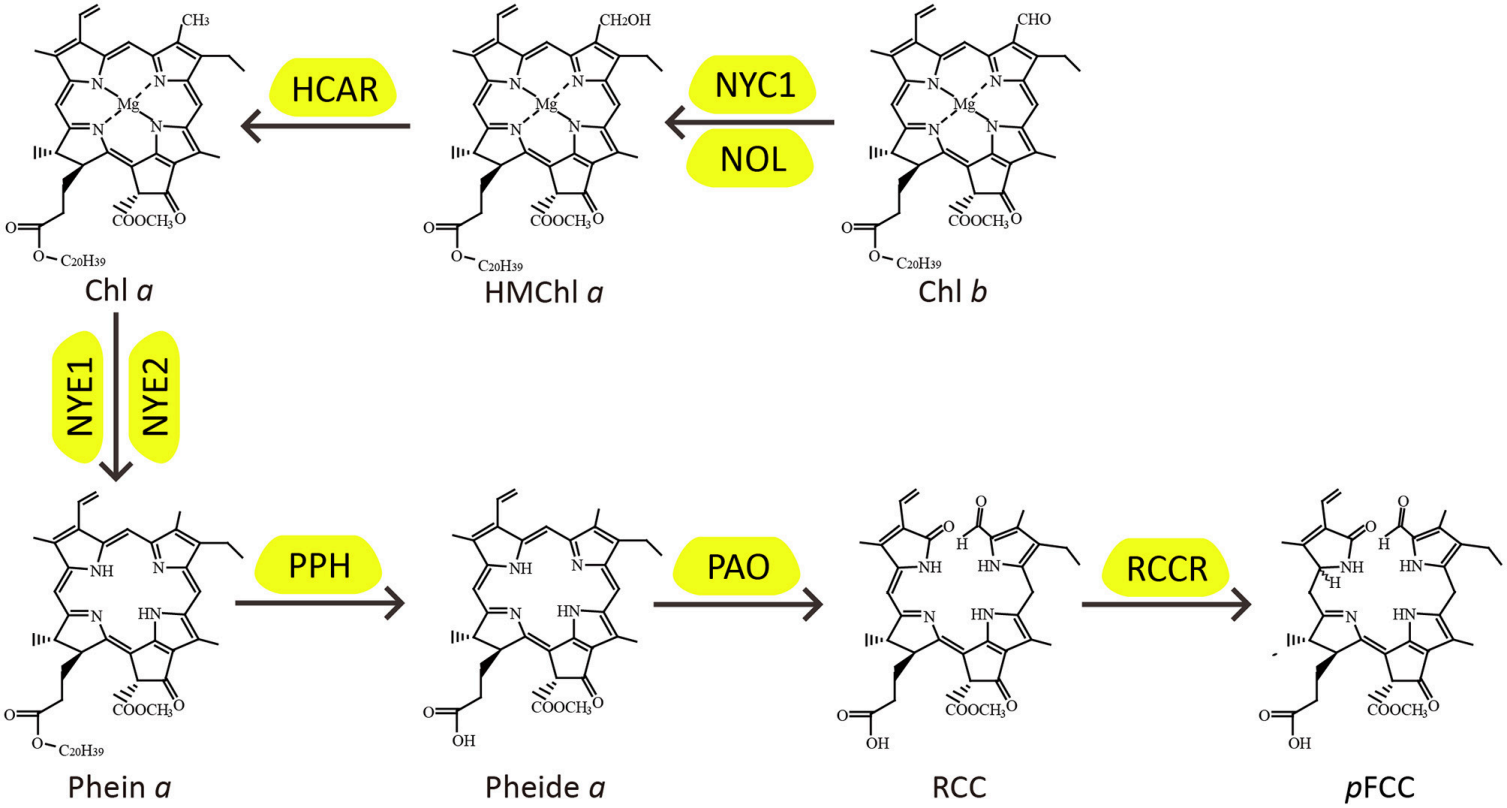
The PAO pathway of chlorophyll degradation.

Chlorophyll (Chl) molecules are synthesized almost instantly upon light exposure of seedlings for harvesting light energy to drive photosynthesis in green organs, and during the processes of leaf senescence, fruit ripening, and seed maturation, they are degraded rapidly, a process called degreening, to facilitate nutrient remobilization and, in some cases, vitamin biosynthesis (Christ and Hörtensteiner, 2014; Vom Dorp et al., 2015). Chl degradation is in fact imperative to plant development for its detoxifying the photo-toxicity of Chl molecules once they are freed from their binding proteins (Hörtensteiner, 2006; Li et al., 2017). Over the last decade or so, the major biochemical pathway of Chl degradation has been revealed by cloning and function analysis of *Chl catabolic genes* (*CCGs*). Because of an important role of the pheophorbide *a* oxygenase (PAO) in Chl degradation, this pathway is designated as PAO pathway (Christ and Hörtensteiner, 2014; Figure 1).

In higher plants, there are two forms of Chl molecules, Chl *a* and Chl *b*. Chl *a* is the degradable form of Chls, and, during leaf senescence, Chl *b* is converted to Chl *a* by Chl *b* reductase [CBR, encoded by *NON-YELLOW COLORING 1* (*NYC1*) and *NYC1-LIKE* (*NOL*)] and 7-hydroxymethyl Chl *a* reductase (HCAR) (Kusaba et al., 2007; Horie et al., 2009; Sato et al., 2009; Meguro et al., 2011). For Chl *a* degradation, Magnesium is initially removed to convert Chl *a* to pheophytin *a* (Phein *a*) by Magnesium-dechelatase, encoded by Mendel's green cotyledon genes, *NON-YELLOWINGs*/*STAY-GREENs* (*NYEs*/*SGRs*) (Armstead et al., 2007; Ren et al., 2007; Chen et al., 2016; Shimoda et al., 2016; Wu et al., 2016). Phein *a* is then hydrolyzed by pheophytinase (PPH) to produce pheophorbide *a* (Pheide *a*) and phytol (Morita et al., 2009; Schelbert et al., 2009; Ren et al., 2010). Remarkably, the green color of Chl catabolites is completely lost when the porphyrin ring of Pheide *a* is cleaved by PAO, resulting in oxidized red Chl catabolite (RCC), which is subsequently catalyzed by red Chl catabolite reductase (RCCR) to generate primary fluorescent Chl catabolite (*p*FCC) (Wüthrich et al., 2000; Pružinská et al., 2003; Pruzinská et al., 2007; Tanaka et al., 2003; Yao and Greenberg, 2006). Finally, the *p*FCC is modified and transported into the vacuole, and isomerized to non-fluorescent products by acidic pH (Christ et al., 2012, 2013; Hauenstein et al., 2016).

Phytohormones and environmental factors have long been known to regulate Chl degradation (Lim et al., 2007); however, the molecular mechanisms involved in these regulations remains largely unknown. In last few years, the success in revealing the biochemical pathway of Chl degradation has led to a rapid progress in elucidation of the molecular mechanisms. Particularly, substantial progress has been made on elucidation of the regulatory roles of ethylene, abscisic acid (ABA), jasmonic acid (JA), and light signaling components on Chl degradation, and a number of regulatory factors of *CCGs* have been identified by using the methods of biochemistry, genetics, and bioinformatics (Delmas et al., 2013; Liang et al., 2014; Sakuraba et al., 2014, 2016; Song et al., 2014; Qiu et al., 2015; Zhang et al., 2015; Zhu et al., 2015; Gao et al., 2016; Ghandchi et al., 2016; Li et al., 2016; Oda-Yamamizo et al., 2016; Yin et al., 2016; Chen et al., 2017; Mao et al., 2017; Table 1). These advances provide some valuable insight into the complexity of the molecular mechanism of hormone- and light-regulated Chl degradation. Here, we review recent progress in this field and discuss important yet unresolved questions regarding the roles and mechanisms of phytohormones and environmental factors in Chl degradation regulation.

**Table 1:** The direct regulatory factors of *Chl catabolic genes* (*CCGs*).

**Species**	**Accession numbers**	**Regulatory factors**	**Signaling**	**Phenotypes of mutants**	**Target *CCGs***	**References**
*Arabidopsis thaliana*	At3g20770	EIN3	Ethylene	Stay-green during leaf senescence	*NYC1, NYE1, PAO*	Qiu et al., 2015
*Arabidopsis thaliana*	At5g39610	ORE1	Ethylene	Stay-green during leaf senescence	*NYC1, NOL, NYE1, PAO*	Qiu et al., 2015
*Citrus sinensis*	Ciclev10010348m	CitERF13^a^	Ethylene	NA	*CitPPH*	Yin et al., 2016
*Arabidopsis thaliana*	At1g34180	ANAC016	Abscisic acid	Stay-green during leaf senescence	*NYE1*	Sakuraba et al., 2016
*Arabidopsis thaliana*	At1g45249/ At4g34000/ At3g19290	ABF2/3/4	Abscisic acid	Stay-green during leaf senescence	*NYC1, NYE1, NYE2, PAO*	Gao et al., 2016
*Arabidopsis thaliana*	At3g24650	ABI3	Abscisic acid	Stay-green during seed maturation	*NYE1, NYE2*	Delmas et al., 2013
*Arabidopsis thaliana*	At1g16540	ABI5	Abscisic acid	Stay-green during leaf senescence	*NYC1, NYE1*	Sakuraba et al., 2014
*Arabidopsis thaliana*	At1g30230	EEL	Abscisic acid	Stay-green during leaf senescence	*NYC1, NYE1*	Sakuraba et al., 2014
*Oryza sativa*	Os03g0327800	OsNAP^b^	Abscisic acid	Accelerated yellowing during leaf senescence^c^	*OsSGR, OsNYC1, OsNYC3, OsRCCR1*	Liang et al., 2014
*Oryza sativa*	Os04g0460600	OsNAC2^d^	Abscisic acid	NA	*OsSGR, OsNYC3*	Mao et al., 2017
*Arabidopsis thaliana*	At1g32640/ At5g46760/ At4g17880	MYC2/3/4^e^	Jasmonic acid	Stay-green during leaf senescence	*NYC1, NYE1, PAO*	Zhu et al., 2015
*Arabidopsis thaliana*	At1g52890/ At3g15500/ At4g27410	ANAC019/055/072^f^	Jasmonic acid	Stay-green during leaf senescence	*NYC1, NYE1, NYE2*	Zhu et al., 2015
*Arabidopsis thaliana*	At2g43010	PIF4	Light	Stay-green during leaf senescence	*NYE1*	Song et al., 2014
*Arabidopsis thaliana*	At3g59060	PIF5	Light	Stay-green during leaf senescence	*NYE1, NYC1*	Zhang et al., 2015
*Arabidopsis thaliana*	At2g45660	SOC1^g^	Light	Accelerated yellowing during leaf senescence	*NYE1, PPH*	Chen et al., 2017

a *Transient over-expression of AtERF17 and SlERF16, which are the homologs of CitERF13 in Arabidopsis and tomato, can lead to Chl degradation in Nicotiana tabacum leaves (Yin et al., 2016)*.

b *The null mutant of AtNAP has a significant stay-green phenotype during leaf and silique senescence (Guo and Gan, 2006; Kou et al., 2012)*.

c *The prematurely senile 1 (ps1-D) is a gain-of-function mutant of OsNAP (Liang et al., 2014)*.

d *OsNAC2 is a rice ortholog of ORE1/ANAC092/AtNAC2 (Mao et al., 2017)*.

e *Over-expression of OsMYC2 significantly promote Chl degradation during leaf senescence in rice (Uji et al., 2017)*.

f *Transient over-expression of oilseed rape BnaNAC55 (Brassica napus L.) lead to a significant decrease in Chl content in Nicotiana benthamiana leaves (Niu et al., 2016)*.

g *SOC1 is a negative regulator of Chl degradation during leaf degreening and senescence (Chen et al., 2017)*.

## The molecular mechanism of ethylene signaling-mediated chl degradation

Ethylene is an important phytohormone, regulating diverse aspects of plant growth and development, especially leaf degreening and fruit ripening (Burg, 1973; Grbic and Bleecker, 1995; Lim et al., 2007; Qiu et al., 2015; Yin et al., 2016). During leaf degreening, the expression of ethylene biosynthetic genes encoding *1-Aminocyclopropane-1-carboxylic acid (ACC) synthase* (*ACS*) and *ACC oxidase* (*ACO*) were significantly up-regulated, and the endogenous ethylene level increased accordingly (van der Graaff et al., 2006; Breeze et al., 2011). *ACO1* antisense tomato plants synthesized less ethylene and delayed leaf degreening (John et al., 1995). *ACSs* octuple mutant, producing ~10% of ethylene in WT, significantly delayed leaf degreening in *Arabidopsis* (Tsuchisaka et al., 2009). Exogenous application of ethylene could induce leaf degreening, whereas treatment with ethylene inhibitors could delay leaf degreening (Serek et al., 1995; Jing et al., 2005). The leaves of *etr1-1*, the mutant of ethylene receptor gene *ETR1*, cannot respond to ethylene treatment and shows a stay-green leaf phenotype (Bleecker et al., 1988; Grbic and Bleecker, 1995; Chao et al., 1997). Consistently, ectopic expression of a mutant form of the *Arabidopsis* ethylene receptor gene *ETR1-1* delayed leaf Chl degradation in *Nicotiana tabacum* (Yang et al., 2008). ETHYLENE INSENSITIVE 2 (EIN2) and its downstream target EIN3 are key components of ethylene signaling, and the mutants of both *EIN2* and *EIN3* exhibit a severe stay-green phenotype during leaf senescence (Chao et al., 1997; Oh et al., 1997). EIN3 positively regulates *ORE1* and *NAP*, the two important regulatory genes of senescence, either directly or indirectly via negatively regulating *miR164*, which in turn cleaves the transcript of *ORE1* (Kim et al., 2009, 2014; Li et al., 2013). These reports convincingly demonstrate that ethylene signaling regulates the pathway of Chl degradation.

Recently, Qiu et al. (2015) reported that the expression of *NYC1, NYE1*, and *PAO* was significantly induced by ethylene treatment in the leaves of *Arabidopsis*, whereas largely repressed in *ein3 eil1* double mutant. The electrophoretic mobility shift assay (EMSA) and dual-luciferase reporter assay demonstrated that EIN3 protein could directly bind to the EBS (EIN3 binding site, AC/TGA/TAC/TCT) in the promoters of *NYC1, NYE1*, and *PAO*, and enhance their promoter activity in *Arabidopsis* protoplasts. Therefore, EIN3 is a positive regulator of ethylene-mediated Chl degradation. Moreover, ORE1, the direct target of EIN3, could bind to the promoters of *NYE1, NYC1, NOL*, and *PAO*, and positively regulate their expression. Intriguingly, EIN3 and ORE1 could promote *NYE1* and *NYC1* expression in an additive manner (Qiu et al., 2015). This progress indicates that EIN3 and EIL1 constitute a major regulatory node of ethylene-triggered degreening, with EIN3 either directly or indirectly regulating the expression of *CCGs*. Notably, Yin et al. (2016) recently revealed that CitERF13, an ethylene responsive factor, could bind to *CitPPH* promoter and positively regulate its expression during citrus fruit degreening (Table 1).

## The molecular mechanism of ABA signaling-mediated chl degradation

ABA can be induced by age-dependent senescence or environmental stresses, such as drought, heat, and salt, and the increase of endogenous ABA level or the exogenous application of ABA accelerates chlorosis and senescence of plant leaves (Raab et al., 2009; Yang et al., 2014; Takasaki et al., 2015; Liu et al., 2016). ABA has therefore long been recognized as a positive regulator of degreening during leaf senescence in plants. It was reported that ABA accelerates leaf degreening and senescence via an AtNAP-SAG113 (a PP2C family protein phosphatase) regulatory module that is involved in the regulation of the stomata movement (Zhang and Gan, 2012).

With an attempt of investigating the direct regulation of *CCGs*, Gao et al. (2016) initially identified ABF3 as a transcriptional regulator of *NYE1* by yeast one-hybrid (Y1H) screening. Further *in vitro* and *in vivo* analyses indicated that ABF2/3/4 directly bind to the promoter of *NYE1*, and up-regulate its transcription. Notably, ABF2/3/4 also bind to the promoters of *NYE2, NYC1*, and *PAO*, and up-regulate their transcription. Under ABA treatment, detached leaves of *abf2 abf3 abf4* triple mutants exhibited an obvious stay-green phenotype, while those of *ABF4-OE* transgenic lines showed an accelerated yellowing phenotype (Gao et al., 2016). ABI5 and EEL, two ABA signaling-related transcription factors, were also found to positively regulate the transcription of *NYE1* and *NYC1* by binding to their promoters (Sakuraba et al., 2014). Similarly, ANAC016, a senescence-associated NAC transcription factor, directly bind to the promoter of *NYE1* and up-regulate its transcription. Leaves of *anac016* mutant showed a stay-green phenotype, while *ANAC016-OX* line displayed an early leaf yellowing phenotype. Interestingly, it indirectly activates *ABSCISIC ALDEHYDE OXIDASE3* (*AAO3*), an ABA biosynthesis gene, via a mediation of NAP (Kim et al., 2013; Yang et al., 2014; Sakuraba et al., 2016). Liang et al. (2014) found that ABA-induced leaf yellowing and senescence were mediated by OsNAP in rice. Unlike AtNAP, OsNAP was specifically induced by ABA but not ethylene. OsNAP directly bind to the promoters of *OsSGR, OsNYC1, OsNYC3* (*PPH*), and *OsRCCR1*, and up-regulated their transcription in rice. Recently, Mao et al. (2017) reported that OsNAC2 could directly bind to the promoters of *OsSGR* and *OsNYC3*, and activate their expression during ABA-induced leaf yellowing and senescence in rice.

ABA also regulates seed maturation. During the processes of seed maturation and embryo degreening, a B3 domain transcription factor ABI3 directly binds to the promoters of *NYE1* and *NYE2*, and up-regulates their transcription, consequently promoting Chl degradation in embryos. Intriguingly, the role of ABI3 in Chl degradation is seed-specific, as the mutant of *ABI3* (*abi3-6*) does not show a stay-green leaf phenotype in the dark (Delmas et al., 2013). This progress has shed a light on the complex molecular mechanism underlying ABA-regulated Chl degradation (Table 1).

## The molecular mechanism of JA signaling-mediated chl degradation

Jasmonic acid is a phytohormone essential for the regulation of multiple developmental processes, including leaf degreening and senescence (Wasternack and Hause, 2013). Ueda and Kato (1980) firstly found that methyl jasmonate (MeJA) could induce leaf degreening in oats. Subsequently, this phenomenon was confirmed in various plant species such as *Arabidopsis*, wheat, rice, and maize (Beltrano et al., 1998; He et al., 2002; Shan et al., 2011; Yan et al., 2012; Lee et al., 2015). Mutants defective for JA synthesis exhibited delayed leaf degreening phenotype (Castillo and León, 2008; Schommer et al., 2008; Yan et al., 2012). COI1-JAZ complex is the co-receptor of JA (Sheard et al., 2010), and the leaves of *coi1* mutant exhibit a stay green phenotype upon MeJA treatment (He et al., 2002; Shan et al., 2011; Lee et al., 2015). MYC2/3/4 could interact with JAZ, acting as the transcriptional activators in JA signaling, whereas bHLH03/13/14/17 were identified as the transcriptional repressors, repressing JA responses. Both MYC2/3/4 and bHLH03/13/14/17 could bind to the promoter of *SAG29*, and activate or repress the expression of *SAG29* during JA-induced leaf senescence (Qi et al., 2015).

In a study of identifying the transcriptional regulators of *CCGs*, Zhu et al. (2015) revealed MYC2 as a putative trans-regulator of *PAO* by using the Y1H screening. MYC2 and its two homologs, MYC3 and MYC4, were then demonstrated to directly bind to the G-box (CACGTG) in the promoters of *PAO, NYC1*, and *NYE1*, and up-regulate their expression during JA-induced Chl degradation. The leaves of *myc2 myc3 myc4* triple mutant showed a stay-green phenotype, whereas those of MYC2/3/4 overexpression lines displayed an accelerated yellowing phenotype upon MeJA treatment. Intriguingly, ANAC019/055/072, the immediate targets of MYC2/3/4 (Bu et al., 2008; Zheng et al., 2012), could also directly up-regulate the expression of *NYE1, NYE2*, and *NYC1*. The triple mutant of *anac019 anac055 anac072* showed a similar stay-green phenotype as *myc2 myc3 myc4* upon MeJA treatment. Moreover, the MYC2 and ANAC019 could interact with each other, and synergistically enhance *NYE1* expression in *Arabidopsis* protoplasts. These findings indicate a hierarchical and coordinated regulatory network during JA-induced Chl degradation (Zhu et al., 2015; Table 1).

## The molecular mechanism of light signaling in regulating chl degradation

Light is the vital environmental factor for plant growth and development. Dark treatment, a simple and effective way for light deprivation, is widely used for studying leaf senescence and degreening (Ren et al., 2007; Christ and Hörtensteiner, 2014). phyB is a red light receptor (Schäfer and Bowler, 2002), and seedlings or mature leaves of *phyB* mutant yellow faster, whereas *PHYB-OX* plants yellow slower than those of WT during dark incubation (Sakuraba et al., 2014). phyB represses PIF4 and PIF5 at the post-transcriptional level (Leivar et al., 2008; Shin et al., 2009). In the dark, leaves of *pif4, pif5*, and *pif1 pif3 pif4 pif5* quadruple mutants all show stay-green phenotypes, while those of *PIF4-OX* and *PIF5-OX* lines show early-yellowing phenotypes (Sakuraba et al., 2014). ELF3 inhibits leaf degreening and senescence by repressing *PIF4* and *PIF5* at the transcriptional level (Nusinow et al., 2011; Sakuraba et al., 2014). After incubating in darkness, leaves of *elf3* senesced faster and leaves of *ELF3-OX* senesced slower than those of WT (Sakuraba et al., 2014). These findings collectively suggest that red light signaling is involved in the regulation of leaf degreening and senescence, with PIF4 and PIF5 acting as key mediators.

Both PIF4 and PIF5 associate with the promoters of *ABI5* and *EEL*, two bZIP family transcription factors, and up-regulate their transcription (Sakuraba et al., 2014). Interestingly, PIF4, PIF5, ABI5, and EEL, as well as EIN3, can all activate the expression of *ORE1*, which encodes an important senescence-promoting transcription factor, by directly binding to its promoter. Meanwhile, ABI5 and EEL could directly activate *NYE1* and *NYC1* by binding to their promoters (Sakuraba et al., 2014). It was further demonstrated that PIF4 directly bind to the promoter of *NYE1*, and PIF5 to the promoters of *NYE1* and *NYC1* to up-regulate their transcription (Song et al., 2014; Zhang et al., 2015). Under dark treatment, endogenous ethylene level was significantly reduced in the leaves of *pif4* mutant, while elevated in those of *PIF4-OX* lines. When treated with ethylene, mutants of *pif3, pif4*, and *pif5* showed stay-green phenotypes, suggesting that PIF3/4/5 play roles in leaf degreening mediated by ethylene signaling (Song et al., 2014).

Recently, in a study designed for exploring the transcriptional regulation of *PPH*, Chen et al. (2017) demonstrated that SUPPRESSOR OF OVEREXPRESSION OF CO 1 (SOC1), a flowering pathway integrator, associates with the promoter of *PPH*, and negatively regulates its transcription. Under dark treatment, leaves of *soc1-6* mutant yellowed earlier, whereas those of *iSOC1-OE* lines partially stayed green, in comparison to their respective controls. Moreover, SOC1 also negatively regulates *NYE1* and *SAG113* at the transcriptional level during dark-induced leaf degreening and senescence. Notably, SOC1 is the only negative regulator of Chl degradation identified so far (Table 1).

## Conclusion and perspectives

Chl degradation is an active and progressive process which is regulated by diverse developmental and environmental clues, and mainly mediated by phytohormones' signaling. In *Arabidopsis*, ethylene signaling promotes leaf degreening through the transcriptional regulation of major *CCGs* by both EIN3 and ORE1, while in citrus fruits by CitERF13 (Qiu et al., 2015; Yin et al., 2016). The severe stay-green phenotype of the mutants of both *EIN3/EIL1* and *ORE1* implies that ethylene signaling is likely the major signaling pathway in regulating degreening during developmental leaf senescence (Kim et al., 2009; Li et al., 2013). ABA signaling mediates Chl degradation at the transcriptional level mainly by ABI3 during seed maturation, whereas, during leaf senescence, by ABI5, EEL, and ABF2/3/4 as well as ANAC016 in *Arabidopsis*, and by OsNAP and OsNAC2 in rice (Delmas et al., 2013; Liang et al., 2014; Sakuraba et al., 2014, 2016; Gao et al., 2016; Mao et al., 2017). Interestingly, these transcription factors have long been known to regulate drought stress/circadian clock (Sanchez et al., 2011), indicating that ABA signaling might be mainly involved in the regulation of leaf degreening-triggered by abiotic stresses. JA signaling directly regulates leaf degreening by MYC2/3/4 and ANAC019/055/072 (Zhu et al., 2015). Considering that the MYCs and ANACs are also involved in the regulation of defense responses, JA signaling likely mediates the degreening process incurred by biotic stresses. Light signal, on the other hand, inhibits leaf degreening by both maintaining the transcription of *SOC1* and repressing the transcription of *PIFs/*reducing PIFs protein accumulation (Sakuraba et al., 2014; Song et al., 2014; Zhang et al., 2015; Chen et al., 2017). Intriguingly, major hormones share their signaling components with light, as loss-of-function mutations of major hormone signaling components (EIN2, EIN3/EIL1, ABI5, EEL, NAP, ORE1, etc.) block light signaling in regulating degreening, causing stay-green phenotypes upon light deprivation, whereas loss-of-functions of major light signaling components, PIFs, also interfere major hormone (e.g., ethylene) signaling in the promotion of degreening (Oh et al., 1997; Guo and Gan, 2006; Li et al., 2013; Kim et al., 2014; Sakuraba et al., 2014; Song et al., 2014).

Although, substantial progress has been made in exploring the molecular regulation of Chl degradation, numerous issues still await to be addressed. (1) There appears to be a “developmental window” for hormone-induced Chl degradation. Ethylene, for example, cannot readily induce leaves to degreen at their young age, and only after a certain developmental stage will leaves allow ethylene to induce their degreening (Jing et al., 2005). What is the molecular basis for the “window effect”? (2) As an inhibitor of Chl degradation, light signal is present during ethylene-, ABA-, and JA-induced or age-dependent leaf degreening (Qiu et al., 2015; Zhu et al., 2015; Gao et al., 2016), but how ethylene, ABA, or JA signaling antagonize light signaling to trigger Chl degradation? (3) There are enormous cross-talks among different hormone signaling pathways which are interweaved with light signaling in the regulation of Chl degradation. It was reported that *ein3* exhibited a stay-green phenotype during MeJA treatment (Li et al., 2013), and *jaz7* showed an early yellowing phenotype under dark treatment (Yu et al., 2016). More work need to be done to elucidate those cross-talks. (4) In addition to ethylene, ABA, and JA, other phytohormones are also found to be involved in the regulation of Chl degradation, with salicylic acid and brassinolide acting as promoters (Morris et al., 2000; Jeong et al., 2010), whereas cytokinin and gibberellic acid as repressors (Fletcher and Osborne, 1966; Lara et al., 2004; Kim et al., 2006). Yet, their regulatory pathways or networks are largely unexplored. (4) Thus far, studies on Chl degradation regulation mainly focus on the transcriptional level. Further investigations need to be extended to post-transcriptional levels, including the translational regulation and post-translational modification. It has been reported that PAO could be interconverted between phosphorylated and dephosphorylated status (Chung et al., 2006).

## Author contributions

All authors listed have made a substantial, direct, and intellectual contribution to the work, and approved it for publication.

### Conflict of interest statement

The authors declare that the research was conducted in the absence of any commercial or financial relationships that could be construed as a potential conflict of interest.
